# Sex differences in friendships and loneliness in autistic and non-autistic children across development

**DOI:** 10.1186/s13229-023-00542-9

**Published:** 2023-02-24

**Authors:** Natalie Libster, Azia Knox, Selin Engin, Daniel Geschwind, Julia Parish-Morris, Connie Kasari

**Affiliations:** 1 Department of Education, UCLA, 457 Portola Plaza, Los Angeles, CA 90024 USA; 2 Center for Autism Research and Treatment, Semel Institute for Neuroscience, UCLA, 760 Westwood Plaza, Los Angeles, CA 90024 USA; 3 Center for Autism Research, Children’s Hospital of Philadelphia, 2716 South St, Philadelphia, PA 19104 USA; 4 David Geffen School of Medicine, UCLA, 10833 Le Conte Ave, Los Angeles, CA 90024 USA; 5 Department of Psychology, University of Pennsylvania, 3720 Walnut St, Philadelphia, PA 19104 USA

**Keywords:** Autism spectrum disorder, Friendships, Loneliness, Developmental differences

## Abstract

**Background:**

Autistic children have been shown to have less complete definitions of friendships and higher levels of loneliness than their non-autistic peers. However, no known studies have explored sex differences in autistic children’s understanding of friendships and reported loneliness across development. Autistic girls demonstrate higher levels of social motivation than autistic boys and appear to “fit in” with their peers, but they often have difficulty recognizing reciprocal friendships during middle childhood. As autistic girls develop a more complex understanding of friendship during adolescence, they may begin to redefine their friendships and experience heightened loneliness. Here, we explored how autistic and non-autistic boys and girls define the meaning of friendship and report feelings of loneliness across development. We also examined their perceptions of friendships and loneliness.

**Methods:**

This mixed-methods study analyzed the transcribed clinical evaluations of 58 autistic children (29 girls) matched to 42 non-autistic children (21 girls) on age and IQ. Transcripts were coded for four categories that children used to define friendships—personality, companionship, dependability, and intimacy—and for reported loneliness. We then compared these codes across diagnosis, sex, and age. Content analyses were further implemented to gain a more holistic understanding of children’s perceptions of friendships and loneliness.

**Results:**

Girls, regardless of diagnosis, were more likely than boys to refer to personality when defining the meaning of friendship, and the likelihood of referring to dependability and intimacy increased with age. Most children reported having at least one friend, though some autistic adolescents reported not having friends or were uncertain whether they had friends. While autistic and non-autistic boys and girls were equally likely to report feeling lonely *at times*, several autistic girls and boys reported being frequently lonely.

**Limitations:**

This study was a secondary data analysis. The standardized set of questions on the ADOS limited the amount of information that children provided about their friendships and perceptions of loneliness.

**Conclusion:**

As with non-autistic children, autistic children acquire a more complex understanding of friendship throughout development. However, as children begin to prioritize dependability and intimacy in friendships, autistic adolescents may have difficulty developing friendships characterized by these constructs. Furthermore, the quantity and/or quality of autistic children’s friendships may not be sufficient to alleviate loneliness.

## Introduction

Many autistic children have the desire to form relationships with others, but often have difficulty developing and maintaining friendships [1–6]. Autism spectrum disorder (ASD) is a neurodevelopmental condition marked by challenges in social interaction and communication, difficulties with peer relationships, and restricted, repetitive behaviors, interests, and activities [7, 8]. Autistic children, both in middle childhood and adolescence, tend to have fewer friends than their non-autistic peers [1, 4], and compared to friendships reported by non-autistic children, those reported by autistic children are less likely to be reciprocated by the nominated friend [3, 9–12]. Furthermore, compared to their non-autistic peers, autistic children consistently report their best friendships to be of lower quality on the friendship qualities scale (FQS) [13], specifically on the subscales of companionship, security-intimacy, closeness, and help [3]. A theoretical explanation for the lower levels of friendship reciprocity and quality observed among autistic children is Milton’s [14] “double empathy problem”. This view emphasizes that both non-autistic and autistic individuals experience equal difficulty understanding the thoughts, feelings, and needs of one another. Compared to non-autistic dyads, autistic children *and* their best friends rate their mutual friendship to be lower in closeness, security-intimacy, and help on the FQS [15], thereby supporting Milton’s theory of mutually reduced empathy.

### Friendship across development

Researchers have explored the developmental trajectory of friendship among non-autistic children by categorizing friendships into three functional roles: companionship (e.g., spending time together in shared play or activities), affection (e.g., mutual liking and caring), and intimacy (e.g., sharing each other’s thoughts, feelings, and experiences) [16–18]. While early friendships tend to be predominantly based on companionship and playmate activities [19–21], young children also demonstrate mutual affection and concern for their friends [21–23]. Meanwhile, intimacy in friendships develops throughout adolescence as children begin to rely on their friends for emotional support [16]. A seminal study by Bauminger and Kasari [24] explored how autistic children define the meaning and purpose of friendships. The researchers found that when autistic and non-autistic children were asked “Can you tell me what a friend is?”, autistic children were less likely than non-autistic children to refer to companionship (59% vs. 89.5%), affection (41% vs. 73.7%), and intimacy (40.9% vs. 68.4%) in their responses. Autistic children were also significantly less likely than non-autistic children to incorporate all three constructs in their definitions of friendship. Participants in the study were in middle childhood and adolescence (7–14 years), yet it is unclear whether developmental status played a role in how children defined the meaning of friendship. Autistic and non-autistic children were more likely to refer to companionship in their definitions than affection and intimacy, suggesting that some (perhaps younger) children may not have had a comprehensive understanding of the role affection and intimacy can play in friendships. Therefore, when autistic and non-autistic children define the meaning of friendship, both diagnostic status *and* age may be influencing factors.

### Sex differences in the quality and meaning of friendships

Studies exploring friendships in autistic children have focused predominantly on males, which is expected given the 4:1 male-to-female ratio observed in autism [7]. Autistic girls are underdiagnosed and remain under-represented in research [6]. However, it is well established that autistic girls demonstrate higher levels of social motivation than autistic boys, increasing their opportunities for group acceptance. For example, observational studies of autistic children in middle childhood reveal that autistic girls tend to stay in close proximity to their peers during free play, optimizing their social opportunities, whereas autistic boys tend to play alone away from peers [25]. The social motivation of autistic girls is further reflected in their use of language. Autistic girls in middle childhood and adolescence are more likely than autistic boys to talk about social groups in conversations [26] and to refer to friends during semi-structured interviews [27]. Several studies report that the *apparent* social success of autistic girls is due to their ability to engage in masking behaviors that conceal potential communication challenges [25, 28, 29]. Autistic girls frequently copy the social behaviors of their non-autistic peers, thereby “masking” their social difficulties [25]—but although autistic girls *appear* to fit in with their peers, they often have difficulty recognizing reciprocal friendships in middle childhood. Studies have found that the friendships of autistic girls are less likely than those of non-autistic girls to be reciprocated by the nominated friend [30]. In a study by Chamberlain et al. [10], a mother of a second-grade girl explained that her daughter had reported having “lots of friends”—however, during recess, she would play by herself in proximity to her peers.

As with non-autistic children, autistic children also seem to develop a more complex understanding of friendship as they enter adolescence. Regardless of diagnosis, adolescent girls emphasize the importance of shared conversations and emotional support in friendships, whereas adolescent boys emphasize the importance of shared activities and practical support [6]. Studies have also revealed that autistic girls produce higher ratings of friendship quality [6, 31] compared to autistic boys during adolescence. Differences in friendship quality between autistic girls and boys may be due to the nature of these relationships, as discussed above. However, these studies have also found that autistic girls produce lower ratings of friendship quality [6, 31] compared to non-autistic girls during adolescence. Therefore, while adolescent autistic and non-autistic girls both understand the role of emotional support and intimacy in friendships [6], autistic girls may be less likely to experience these constructs in their own friendships.

### Loneliness in autistic children and adolescents

Lower levels of friendship reciprocity and quality are associated with greater loneliness in non-autistic and autistic children [32–34]. Loneliness in autistic children is associated with lower levels of self-worth [32], heightened levels of social anxiety [35], and a higher likelihood of depression in adolescence [36, 37]. Studies have found that reported loneliness among autistic children increases across development. Autistic and non-autistic children in second-through fifth-grade classrooms report similar levels of loneliness [10]. However, among samples that include participants in middle childhood and adolescence, autistic children report higher rates of loneliness than their non-autistic peers [1, 24, 32, 34, 38]. Developmental differences in reported loneliness among autistic children may be due to perceived social involvement. Autistic children in middle childhood rate themselves as being more socially involved (i.e., interacting with more peer groups) than what is reported by their peers [10]. Since these children *perceive* themselves as being socially integrated in their classrooms, they may not experience feelings of loneliness. Autistic adolescents are also less socially involved than their non-autistic peers, but unlike autistic children in middle childhood, they may have a more accurate perception of their social integration and report higher rates of loneliness [38].

Loneliness among autistic adolescents has also been shown to be related to their reported number of close friendships and their reported satisfaction with those friendships [39]. Studies have found that most autistic children in middle childhood report being highly satisfied with their friendships [3, 17, 40]. However, autistic adolescents may experience difficulty in developing friendships marked by affection and intimacy, which become increasingly important throughout adolescence [16, 24]. Since autistic adolescents also report their best-friendships to be of lower quality than their non-autistic peers [3, 6, 31], they may experience less satisfaction with these friendships and experience higher rates of emotional loneliness. Seminal research on loneliness conducted by Weiss [41] suggests that there are two types of loneliness: social and emotional. Social loneliness refers to the absence of accessible social networks and peer groups, which provide companionship and a sense of belonging. Meanwhile, emotional loneliness refers to the absence of close, intimate friends or people that one can turn to for support [1, 42].

Studies have found that among samples that include participants in middle childhood and adolescence, autistic children report higher rates of social and emotional loneliness compared to their non-autistic peers [1]. However, as expected, prior studies exploring loneliness in autistic adolescents have focused on males. Since adolescent autistic girls are more socially motivated than autistic boys and report higher levels of friendship quality [6, 31], they may experience lower levels of social and emotional loneliness. Meanwhile, autistic girls report lower levels of friendship quality [6, 31] compared to non-autistic girls and therefore may report higher levels of emotional loneliness. It should be noted that previous research on friendships and loneliness has primarily focused on autistic children and adolescents who are able to use spoken language and are often functioning within an expected range of development for their chronological age. Minimally verbal and intellectually disabled individuals remain under-represented.

### The current study

The current mixed-methods study had three aims. First, we aimed to explore how autistic and non-autistic boys and girls defined the meaning of friendship and then determine whether these definitions differed across sex, ASD diagnostic status, and age. While prior research [24] coded children’s definitions of friendship into three categories—companionship, affection, and intimacy—we qualitatively analyzed children’s definitions to identify additional roles of friendship that children described. Second, we aimed to determine whether reported loneliness among autistic and non-autistic children differed across sex, ASD diagnostic status, and age. Finally, we aimed explore children’s perceptions of friendships and loneliness, specifically if they reported the absence of friendships and if they frequently felt lonely. Similar to previous reports, we limited our investigation to individuals who had spoken language and could complete Module 3 of the autism diagnostic observation schedule (ADOS) [43].

## Methods

### Participants

The current mixed-methods study was a secondary analysis of transcribed clinical evaluations from 100 children between 6 and 15 years—58 autistic children (29 girls) and 42 non-autistic children (21 girls). Participant samples overlap with Libster et al. [44]. The transcribed evaluations were selected from two ongoing studies and five completed studies in which participants were administered the ADOS Module 3 [43]. The number of participants whose data were used from each study is listed in Table 1. Autistic girls were the smallest group in each of the original studies. Therefore, every autistic girl from each of the original studies was included in the current sample to maximize the number of autistic girls. Autistic girls who did not respond to at least 50% of the ADOS questions were then excluded. Afterward, groups of autistic boys and non-autistic boys and girls were created that matched the autistic girls on age and IQ. If a participant did not respond to at least 50% of the ADOS questions, they were replaced with another participant. The UCLA Institutional Review Board (IRB) approved data sharing of all seven primary studies, as did the IRB at each site.

**Table 1:** Primary studies from which current sample was selected

	ASD boys	ASD girls	Non-ASD boys	Non-ASD girls
Ongoing studies				
P50HD055784	2	2	–	–
2R01MH100027-11	4	4	–	–
Completed studies				
Dean et al. [46]	1	1	–	–
Kasari et al. [45]	2	3	–	–
Lord et al. [47]	1	1	–	–
Cola et al. [27]	10	8	17	14
Parish-Morris et al. [48]	9	10	4	7
Total	29	29	21	21

Two ongoing studies (P50HD055784, 2R01MH100027-11) are currently being conducted at the University of California, Los Angeles. Two completed studies [45, 46] took place at UCLA, one completed study [47] took place at both the University of North Carolina, Chapel Hill and the University of Chicago, and the other two completed studies [27, 48] took place at Children’s Hospital of Philadelphia

This study focused on autistic children who were administered Module 3 of the ADOS. Thus, they were children with spoken language (including complex sentences and references to non-present people or events) and who had average to above average IQs. The transcribed evaluations of non-autistic children were selected from the two studies at Children’s Hospital of Philadelphia [27, 48], in which semi-structured behavioral samples of non-autistic children were compared to those of autistic children. In the current study, autistic and non-autistic boys and girls were matched by group (frequency matching) on age (range 6–15 years) and IQ. Two one-way ANOVAs were conducted to test the success of the matching procedure and did not reveal significant differences in age and IQ across the four sex/diagnostic groups (see Table 2). Furthermore, ADOS calibrated severity scores (CSS) were matched across autistic boys and girls and across non-autistic boys and girls. Independent samples *t*-tests were conducted to test the success of the matching procedure and did not reveal significant differences in ADOS severity scores across sex within each diagnostic group. ADOS Social Affect (SA) and Restricted Repetitive Behaviors (RRB) scores also did not differ across sex within each diagnostic group, as tested using independent samples *t*-tests (see Table 2).

**Table 2:** Participant characteristics by sex and diagnostic group

	ASD boys (*n* = 29)	ASD girls (*n* = 29)	*M* (SD)	*p*	Non-ASD boys (*n* = 21)	Non-ASD girls (*n* = 21)	*M* (SD)	*p*	Total *M* (SD)	*p*
Age *M* (SD)	10.48 (1.64)	10.41 (1.97)	10.45 (1.80)	0.89	9.90 (2.64)	9.81 (2.71)	9.86 (2.65)	0.91	10.20 (2.20)	0.62
IQ *M* (SD)	104.14 (14.90)	103.69 (17.09)	103.91 (15.90)	0.92	108.29 (10.83)	105.14 (14.91)	106.71 (12.97)	0.44	105.09 (14.73)	0.72
ADOS CSS *M* (SD)	6.45 (2.25)	6.45 (2.25)	6.38 (2.32)	0.82	1.19 (0.40)	1.29 (0.78)	1.24 (0.62)	0.62	–	–
ADOS SA *M* (SD)	6.69 (2.17)	6.34 (2.33)	6.52 (2.24)	0.56	1.86 (0.91)	1.81 (1.17)	1.83 (1.03)	0.88	–	–
ADOS RRB *M* (SD)	6.14 (3.07)	6.83 (2.22)	6.48 (2.68)	0.33	1.67 (1.71)	1.67 (1.71)	1.67 (1.69)	0.99	–	–

Racial and ethnic demographic information was available for 99 of the 100 participants—68% of participants were White, 19% were Black, 3% were Hispanic, 3% were Asian, and 6% were multiracial. Participants had been administered the ADOS and demonstrated evidence that they understood the assessment questions and could articulate their responses. Children who did not respond to at least 50% of the ADOS interview questions were excluded from the study, as rich and comprehensive qualitative data were needed to conduct the content analyses.

### Measures

In the seven primary studies, the ADOS (Module 3) [43] was administered in a range of settings—clinicians either visited the child’s school or home or the child visited the institution where the study was taking place. Each administration of the ADOS was videotaped for later analysis. The ADOS is a semi-structured diagnostic assessment used to measure behaviors that may be symptomatic of ASD, including challenges in communication, social interaction, and play. After the assessment is finished, the clinician rates a series of items based on the child’s performance and observations made during the assessment [49]. These ratings are used to formulate diagnostic algorithms for two behavioral domains—Social Affect (SA) and Restricted Repetitive Behaviors (RRB). The SA and RRB algorithms are then standardized to provide a measure of overall autism symptom severity, known as the Calibrated Severity Score (CSS) [50, 51]. ADOS severity scores, which range from 1 (least severe) to 10 (most severe), can be used to compare autism symptom severity across children of different ages [51].

Children’s responses to interview questions on the ADOS were coded, with a focus on questions about the nature of friendships and loneliness. For the purposes of this study, responses to the following questions were transcribed and coded: (1) “Do you have any friends? Can you tell me about them?” (2) “What does being a friend mean to you? How do you know if someone is your friend?” (3) “Do you ever feel lonely?” and (4) “Do you think other kids/people your age ever feel lonely?” While the ADOS interview is semi-structured and allows for follow-up questions to children’s responses, clinicians follow a standardized interview protocol and receive standard training on its administration, along with reliability assessments. Most clinicians in the current study followed the protocol. However, one interview excluded the questions about what being a friend means and how to know if someone is a friend, and four interviews excluded the question about perceived loneliness in other people. When this occurred, the child’s response was coded as “missed”. These codes were omitted from the analyses.

#### Defining the meaning of friendship

Responses to the questions “What does being a friend mean to you?” and “How do you know if someone is your friend?” were transcribed, and a manifest content analysis was implemented to code and quantify children’s definitions of friendships. Using the guidelines established by Elo and Kyngäs [52], the transcripts were first reviewed to gain an understanding of the data. Afterwards, children’s definitions of friendships were identified and grouped into categories. A list of codes that represented these categories was then generated and applied to the text through detailed, line-by-line annotations of the transcripts. The coding procedure was implemented using Dedoose software [53].

We identified four content categories that children referred to when defining the meaning of friendship—*personality, companionship, dependability,* and *intimacy. Personality* was coded if the child referred to a friend as someone who has positive qualities (e.g., “Someone who’s very nice to you,” “Someone who helps you”). *Companionship* was coded if the child referred to a friend as someone they like or enjoy being with (e.g., “You like playing with each other,” “Someone who you can just have fun with”). *Dependability* was coded if the child referred to a friend as someone they care about or can rely on (e.g., “Someone who will be there for you,” “When I’m in trouble they’d stand up for me”). Finally, *intimacy* was coded if the child referred to a friend as someone to whom they can disclose their feelings (e.g., “You both can tell each other secrets and confide in each other,” “I can trust them with like secrets and stuff”).

Multiple codes were applied to the text if the child referred to more than one category when describing what friendship meant to them. For example, if the child referred to a friend as someone who was kind to them and who they relied on, the excerpt was coded as *personality* and *dependability*. While *affection* has been used in prior research [24] to refer to mutual liking and caring in friendships, the current study distinguished liking vs. caring. When the child referred to a friend as someone they liked, this was coded as *companionship* (e.g., “They like you and you like to play with each other”). Meanwhile, when the child referred to a friend as someone they cared about, could rely on, or could turn to for support, this was coded as *dependability* (e.g., “You take care of one another”). *Dependability* codes in the current study therefore had stronger emotional connectedness than *companionship* codes. If the child did not refer to *personality, companionship, dependability,* or *intimacy* in their definitions (e.g., “Friend means that you are the best friend in the world"), their response was coded as *incomplete*.

A second coder annotated 25% of the transcripts and interrater reliability was calculated (Cohen’s kappa = 0.85). Four logistic regression analyses were then conducted to test whether sex and/or diagnosis predicted children’s references to personality, companionship, dependability, and intimacy when describing the meaning of friendship. Another logistic regression analysis was conducted to test whether sex and/or diagnosis predicted incomplete definitions of friendship. Finally, a Poisson regression analysis with robust error variance was conducted to test whether sex and/or diagnosis predicted the number of categories (0–4) that children referred to when describing the meaning of friendship. Age and IQ were controlled in all six models, which were conducted using R version 4.1.0.

#### Reported loneliness

Responses to the question “Do you ever feel lonely?” were transcribed and coded for children’s reported loneliness. “Yes” was coded if the child reported that they felt lonely at times, and “no” was coded if the child reported that they never felt lonely. A second coder annotated 25% of the transcripts and interrater reliability was calculated (Cohen’s kappa = 1.00). A logistic regression analysis was then conducted to test whether sex and/or diagnosis predicted perceived loneliness, after controlling for age and IQ. Two responses did not clearly answer the question and were therefore omitted from the analysis. The logistic regression model was conducted using R version 4.1.0.

#### Perceptions of friendships and loneliness

A final manifest content analysis was implemented to explore children’s perceptions of friendships and loneliness, specifically if they reported the absence of friendships and if they frequently felt lonely. Children’s responses to the following questions were transcribed and analyzed: (1) “Do you have any friends? Can you tell me about them?” (2) “Do you ever feel lonely?” and (3) “Do you think other kids/people your age ever feel lonely?”. The transcripts were coded using the previously discussed guidelines established by Elo and Kyngäs [52]. The first round of analysis examined children’s reported friendships. Occurrences in which children reported not having friends or were unable to provide details about friends were coded and counted. These frequency counts were then compared across sex and ASD diagnostic status. The second round of analysis examined the frequency in which children reported feeling lonely. Occurrences in which children referred to being frequently alone were coded and counted. These frequency counts were then compared across sex and ASD diagnostic status. Since the identities of researchers ultimately shape the research process [54], it is important to reflect on our positionality. This research was conducted by White, non-autistic psychologists at academic institutions who have experience conducting research to benefit autistic children and adults.

## Results

### Defining the meaning of friendship

Four separate logistic regression models tested predictors of children’s references to personality, companionship, dependability, and intimacy when defining the meaning of friendship. An additional logistic regression model tested predictors of incomplete definitions of friendship, in which children omitted all four categories in their responses. The interaction between sex and ASD diagnostic status was not a significant predictor of any of the five outcomes and was taken out of the final models (*p* value range 0.15–0.97).

Final models are depicted in Table 3. In the first model, sex was found to be a significant predictor of personality. Girls were 2.51 times more likely than boys to refer to personality when defining the meaning of friendship (*p* = 0.03). Meanwhile, in the second model, neither sex nor ASD diagnostic status was a significant predictor of companionship. Autistic boys and girls were as likely as non-autistic boys and girls to refer to companionship when defining the meaning of friendship. In the third model, age was found to be a significant predictor of dependability. For every 1-year increase in age, children were 1.62 times more likely to refer to dependability when defining the meaning of friendship (*p* < 0.001). In the fourth model, both IQ and age were significant predictors of intimacy. For every one-point increase in IQ, children were 1.08 times more likely to refer to intimacy when defining the meaning of friendship (*p* = 0.03). Furthermore, for every 1-year increase in age, children were 1.83 times more likely to refer to intimacy (*p* = 0.01). Finally, in the fifth model, age was a significant predictor of incomplete definitions of friendship. For every 1-year increase in age, children were 0.45 less likely to omit all four categories—personality, companionship, dependability, and intimacy—when defining the meaning of friendship (*p* = 0.002).

**Table 3:** Final odds ratio estimates for referred categories when defining the meaning of friendship

	Personality	Companionship	Dependability	Intimacy	Incomplete
	OR	OR	OR	OR	OR
	CI	CI	CI	CI	CI
Diagnosis	0.59	1.25	0.44	0.22	2.34
	0.25–1.37	0.55–2.87	0.17–1.10	0.02–1.31	0.56–12.41
Sex	2.51*	1.24	0.64	3.85	1.18
	1.11–5.81	0.55–2.80	0.26–1.52	0.66–3.31	0.33–4.29
IQ	0.99	1.02	1.01	1.08*	0.96
	0.97–1.02	0.99–1.05	0.98–1.05	1.01–1.16	0.91–1.01
Age	0.98	1.12	1.62*	1.83*	0.45*
	0.80–1.19	0.93–1.37	1.29–2.12	1.21–3.23	0.25–0.70

* indicates significant predictor. The reference groups for diagnosis and sex were non-autistic children and boys, respectively

A Poisson regression model with robust error variance further tested predictors of the number of categories that children included in their definitions. The interaction between sex and ASD diagnostic status was not a significant predictor and taken out of the final model. Age was found to be a significant predictor. For every 1-year increase in age, the expected number of categories in children's definitions increased by 10% (95% CI [1.04, 1.16]), all else equal (*p* < 0.001).

### Reported loneliness

To test predictors of reported loneliness, in which the question ““Do you ever feel lonely?” was coded as “yes,” a logistic regression analysis was conducted. The interaction between sex and ASD diagnostic status was not a significant predictor of perceived loneliness and was taken out of the model (*p* = 0.68). Autistic boys and girls were as likely as non-autistic boys and girls to report feeling lonely at times (OR = 2.15, *p* = 0.07).

### Perceptions of friendships and loneliness

When asked if they had friends, five autistic children (three girls) either reported not having any friends or were uncertain whether they had friends (e.g., “I really don’t have a lot of friends,” “I talk to people, but I’m not sure if they’re friends of not since I’m usually quiet at school”). This was not reported by any non-autistic children. The autistic boys and girls who reported not having friends or who were uncertain were all adolescents (12 years of age or older). However, the majority of autistic children in the study reported having at least one friend.

Furthermore, ten autistic children (five girls) reported that they were often lonely (e.g., “I’m alone most of the time,” “At school, nobody wants to play with me”). This was not reported by any non-autistic children. These children only referred to the social dimension of loneliness (i.e., the absence of accessible peer groups), not emotional loneliness (i.e., the absence of close, intimate friends). Interestingly, all the autistic children who reported being frequently lonely also reported having at least one friend.

## Discussion

This study explored how autistic and non-autistic boys and girls defined the meaning of friendship and examined their perceptions of friendships and loneliness. This study further compared children’s definitions of friendships and reported loneliness across sex, ASD diagnostic status, and age. When defining the meaning of friendship, girls, regardless of diagnosis, were more likely than boys to refer to personality (i.e., someone who is nice). This finding may be explained by children’s stereotypes about how boys and girls are “supposed” to behave. Gender stereotypes begin to develop in early childhood [55, 56] and continue throughout adolescence and adulthood [57, 58]. Prescribed gender-typical behaviors for girls and women include being warm, gentle, and sympathetic, while those for boys and men include being dominant, aggressive, and independent [56–59]. As with non-autistic children, autistic children also tend to report having same-sex friends [6, 30]—therefore, both autistic and non-autistic girls may expect their friends to exhibit “girl-typical” behaviors.

Although autistic girls often refer to positive personality traits, such as kindness, when defining the meaning of friendship, they may not necessarily be treated with kindness by their own friends. This was demonstrated by two autistic girls in the current study, who reported being bullied by their friends (“Katie bullies Paige, Freddy, sometimes even me, but she's still my friend”). In another study examining the friendship experiences of autistic adolescents, Sedgewick et al. [5] also found that autistic girls reported high levels of relational aggression within their friendships. Autistic girls may therefore have difficulty identifying inappropriate friendship behaviors. Parents of autistic children, especially girls, may need to engage in discussions on how to identify “true” friends. It is equally important for teachers and school personnel to identify instances of bullying and promote prosocial behaviors among autistic *and* non-autistic children.

Our findings further revealed that autistic and non-autistic boys and girls were equally likely to refer to companionship when defining the meaning of friendship, contrary to the results found by Bauminger and Kasari [24]. The discrepancies between the two studies could be attributed to variations in analyses. The current study used a larger sample (*N* = 100 vs. *N* = 44), defined companionship differently, and implemented regression analyses. Autistic and non-autistic children in the current study were also equally likely to refer to dependability and intimacy when defining the meaning of friendship, controlling for sex, age, and IQ. However, age was a significant predictor of both categories—the likelihood of referring to dependability and intimacy increased with age. These findings are consistent with the literature on the development of friendships. Although young children demonstrate mutual affection and concern for their friends [20–22], they may not be able to express or articulate these feelings until adolescence [24]. Intimacy is an even more complex construct that does not develop until adolescence [16]. In the current study, only seven children—all adolescents—referred to intimacy. Another significant predictor of intimacy was IQ—as IQ increased, the likelihood of referring to intimacy increased.

Age was also a significant predictor of incomplete definitions of friendship, as well as the number of categories that children referred to when defining the meaning of friendship. The likelihood of having incomplete definitions of friendship *decreased* with age, and on a similar note, the expected number of categories that children included in their definitions *increased* with age. Although adolescents in the current study demonstrated more complete definitions of friendships than children in middle childhood, our content analysis revealed that a few autistic adolescents reported not having any friends or were uncertain whether they had friends. Autistic and non-autistic children in middle childhood may be more indiscriminate when choosing friends—for example, they may consider every student in their classroom to be a “friend”. However, as children begin to develop a more complex understanding of friendship, autistic adolescents may experience difficulty in developing close, intimate friendships [3].

Surprisingly, given previous research, autistic boys and girls were as likely as non-autistic boys and girls to report feeling lonely at times, controlling for age and IQ. However, it is important to note that the interview question asked if children *ever* felt lonely—the question did not address the frequency or nature of loneliness. Our content analysis revealed that several autistic boys and girls, in middle childhood and adolescence, reported that they were often lonely. While the autistic children in Bauminger et al.’s study [1] reported high levels of social and emotional loneliness using a rating-scale format, the autistic children in the current study only referred to social loneliness. Therefore, when verbally describing feelings of loneliness, autistic children may be more likely to refer to social rather than emotional loneliness. Interestingly, all the autistic children in the current study who reported being frequently lonely also reported having at least one friend. This suggests that the quantity and/or quality of autistic children’s friendships, which is often lower than that of non-autistic children, may not be sufficient to alleviate loneliness.

### Limitations

This study has many strengths, including a relatively large, well-matched sample of autistic and non-autistic girls and boys. It also has limitations, which we hope will be addressed in future research. While the majority of autistic children in the current study reported having at least one friend, we were unable to examine the quality, reciprocity, or satisfaction of children’s friendships. Future studies should use measures of friendship quality [13], reciprocity [30], and satisfaction [40] to determine whether these components of friendship differ across sex, ASD diagnostic status, and age. Another limitation is that this study was a secondary data analysis of children’s responses to interviews on the ADOS. The standardized set of questions on the ADOS limited the amount of information that children provided about the meaning of friendships and perceptions of loneliness. This presented a major limitation in analyzing children’s responses to the loneliness interview. Due to the phrasing of the question “Do you *ever* feel lonely?”, we were unable to examine the degree to which autistic and non-autistic boys and girls experienced social and emotional loneliness. Future studies should use self-reports that measure the degree of social and emotional loneliness [1] and examine differences in reported loneliness across sex, ASD diagnostic status, and age.

Although the ADOS is a standardized measure, there was still variability in the administration of the assessment across participants. There were variations in obtaining answers to all of the questions, and follow-up questions asked by clinicians may have influenced children’s responses—clinicians who asked more follow-up questions may have elicited more information from children. Furthermore, the current study only included participants who responded to at least 50% of the interview questions. While this was necessary to collect rich qualitative data, we were unable to examine perceived friendships and loneliness in autistic children who did not have the verbal communication skills to talk about their experiences. Future studies should therefore use multi-informant approaches, including parent- and teacher-report, to acquire a more holistic understanding of autistic children’s social experiences. The generalizability of the current study is also limited by race, as the majority of the participants were White.

### Future directions and implications

The current study has important implications for interventions designed to improve peer relationships among speaking autistic children. Prior interventions have focused on the development of social skills in autistic children in middle childhood [45, 60] and adolescence [61, 62]. However, several autistic children in the current study reported the absence of friendships and frequent loneliness despite having successful interactions with their peers. Future interventions may therefore need to also focus on the development of reciprocal, high-quality friendships among autistic children. It is important to note that autistic children often report being excluded by their non-autistic peers [12, 30, 63]. Non-autistic children have been shown to rate their first impressions of autistic children more negatively than their first impressions of other non-autistic children [64], and autistic children often attribute peer rejection to personal attributes, such as being “different” or “not approachable” [65]. Therefore, future interventions should not solely focus on teaching autistic students the social skills to “fit in,” but should also focus on the acceptance of autistic children by their non-autistic peers.

## Conclusion

The current study emphasizes the role of developmental status in autistic children’s definitions of friendship and descriptions of friends. As with non-autistic children, autistic children acquire a more complex understanding of friendship—characterized by personality, companionship, dependability, and intimacy—throughout development. However, autistic children may experience difficulty in developing close, intimate friendships, which become increasingly important throughout adolescence. Having reciprocal, high-quality friendships during childhood and adolescence may protect autistic children from loneliness, which they experience at higher rates than their non-autistic peers. The current study therefore highlights the importance of interventions designed to improve friendship reciprocity, quality, and satisfaction among autistic children and adolescents.

## Data Availability

The datasets generated and/or analyzed during the current study are not publicly available due to privacy concerns for minors with disabilities.

## Abbreviations

ADOS: Autism Diagnostic Observation Schedule-Second Edition
ASD: Autism spectrum disorder
CCS: Calibrated severity score
IQ: Intelligence quotient
M: Mean
OR: Odds ratio
SA: Social affect
SD: Standard deviation
RRB: Restricted repetitive behaviors

## References

[1] Bauminger N, Shulman C (2003). The development and maintenance of friendship in high-functioning children with autism: maternal perceptions. Autism.

[2] Church C, Alisanski S, Amanullah S (2000). The social, behavioral, and academic experiences of children with Asperger syndrome. Focus Autism Other Dev Disabil.

[3] Petrina N, Carter M, Stephenson J (2014). The nature of friendship in children with autism spectrum disorders: a systematic review. Res Autism Spectr Disord.

[4] Rowley E, Chandler S, Baird G, Simonoff E, Pickles A, Loucas T, Charman T (2012). The experience of friendship, victimization and bullying in children with an autism spectrum disorder: associations with child characteristics and school placement. Res Autism Spectr Disord.

[5] Sedgewick F, Hill V, Yates R, Pickering L, Pellicano E (2016). Gender differences in the social motivation and friendship experiences of autistic and non-autistic adolescents. J Autism Dev Disord.

[6] Sedgewick F, Hill V, Pellicano E (2019). ‘It’s different for girls’: gender differences in the friendships and conflict of autistic and neurotypical adolescents. Autism.

[7] Maenner MJ, Shaw KA, Bakian AV, Bilder DA, Durkin MS, Esler A, Furnier SM, Hallas L, Hall-Lande J, Hudson A, Hughes MM, Cogswell ME (2021). Prevalence and characteristics of autism spectrum disorder among children aged 8 years—autism and developmental disabilities monitoring network, 11 sites, United States, 2018. MMWR Surveill Summ.

[8] American Psychiatric Association (2013). Diagnostic and statistical manual of mental disorders.

[9] Kuo MH, Orsmond GI, Cohn ES, Coster WJ (2013). Friendship characteristics and activity patterns of adolescents with an autism spectrum disorder. Autism.

[10] Chamberlain B, Kasari C, Rotheram-Fuller E (2007). Involvement or isolation? The social networks of children with autism in regular classrooms. J Autism Dev Disord.

[11] Kasari C, Locke J, Gulsrud A, Rotheram-Fuller E (2011). Social networks and friendships at school: comparing children with and without ASD. J Autism Dev Disord.

[12] Rotheram-Fuller E, Kasari C, Chamberlain B, Locke J (2010). Social involvement of children with autism spectrum disorders in elementary school classrooms. J Child Psychol Psychiatry.

[13] Bukowski WM, Hoza B, Boivin M (1994). Measuring friendship quality during pre-and early adolescence: the development and psychometric properties of the friendship qualities scale. J Soc Pers Relationsh.

[14] Milton DE (2012). On the ontological status of autism: the ‘double empathy problem’. Disabil Soc.

[15] Bauminger N, Solomon M, Aviezer A, Heung K, Gazit L, Brown J, Rogers SJ (2008). Children with autism and their friends: a multidimensional study of friendship in high-functioning autism spectrum disorder. J Abnorm Child Psychol.

[16] Buhrmester D (1990). Intimacy of friendship, interpersonal competence, and adjustment during preadolescence and adolescence. Child Dev.

[17] Calder L, Hill V, Pellicano E (2013). ‘Sometimes I want to play by myself’: understanding what friendship means to children with autism in mainstream primary schools. Autism.

[18] Howes C, Newcomb AF, Hartup WW, Bukowski WM (1996). The earliest friendships. The company they keep: friendships in childhood and adolescence.

[19] Brownell C, Brown E, Van Hasselt VB, Hersen M (1992). Peers and play in infants and toddlers. Handbook of social development.

[20] Gifford-Smith ME, Brownell CA (2003). Childhood peer relationships: social acceptance, friendships, and peer networks. J Sch Psychol.

[21] Howes C, Laursen B, Rubin KH, Bukowski WM (2011). Friendship in early childhood. Handbook of peer interactions, relationships, and groups.

[22] Dunn J (2004). Children's friendships: the beginnings of intimacy.

[23] Howes C, Lee L, Spodek B, Saracho ON (2006). If you’re not like me, can we play?. Learning in early childhood education.

[24] Bauminger N, Kasari C (2000). Loneliness and friendship in high-functioning children with autism. Child Dev.

[25] Dean M, Harwood R, Kasari C (2017). The art of camouflage: gender differences in the social behaviors of girls and boys with autism spectrum disorder. Autism.

[26] Song A, Cola M, Plate S, Petrulla V, Yankowitz L, Pandey J, Schultz RT, Parish-Morris J (2021). Natural language markers of social phenotype in girls with autism. J Child Psychol Psychiatry.

[27] Cola M, Yankowitz LD, Tena K, Russell A, Bateman L, Knox A, Plate S, Cubit LS, Zampella CJ, Pandey J, Schultz RT (2022). Friend matters: sex differences in social language during autism diagnostic interviews. Mol Autism.

[28] Hiller RM, Young RL, Weber N (2014). Sex differences in autism spectrum disorder based on DSM-5 criteria: evidence from clinician and teacher reporting. J Abnorm Child Psychol.

[29] Hull L, Petrides KV, Mandy W (2020). The female autism phenotype and camouflaging: a narrative review. Rev J Autism Dev Disord.

[30] Dean M, Kasari C, Shih W, Frankel F, Whitney R, Landa R, Lord C, Orlich F, King B, Harwood R (2014). The peer relationships of girls with ASD at school: comparison to boys and girls with and without ASD. J Child Psychol Psychiatry.

[31] Head AM, McGillivray JA, Stokes MA (2014). Gender differences in emotionality and sociability in children with autism spectrum disorders. Mol Autism.

[32] Bauminger N, Shulman C, Agam G (2004). The link between perceptions of self and of social relationships in high-functioning children with autism. J Dev Phys Disabil.

[33] Lodder GM, Scholte RH, Goossens L, Verhagen M (2017). Loneliness in early adolescence: friendship quantity, friendship quality, and dyadic processes. J Clin Child Adolesc Psychol.

[34] Whitehouse AJ, Durkin K, Jaquet E, Ziatas K (2009). Friendship, loneliness and depression in adolescents with Asperger's syndrome. J Adolesc.

[35] White SW, Roberson-Nay R (2009). Anxiety, social deficits, and loneliness in youth with autism spectrum disorders. J Autism Dev Disord.

[36] Baczewski L, Kasari C, Coplan RJ, Bowker JC, Nelson LJ (2021). Loneliness and associated mental health sequelae in individuals with autism spectrum disorder. The handbook of solitude: psychological perspectives on social isolation, social withdrawal, and being alone.

[37] Qualter P, Brown SL, Munn P, Rotenberg KJ (2010). Childhood loneliness as a predictor of adolescent depressive symptoms: an 8-year longitudinal study. Eur Child Adolesc Psychiatry.

[38] Locke J, Ishijima EH, Kasari C, London N (2010). Loneliness, friendship quality and the social networks of adolescents with high-functioning autism in an inclusive school setting. J Res Spec Educ Needs.

[39] Jackson SL, Hart L, Brown JT, Volkmar FR (2018). Brief report: self-reported academic, social, and mental health experiences of post-secondary students with autism spectrum disorder. J Autism Dev Disord.

[40] Petrina N, Carter M, Stephenson J, Sweller N (2017). Friendship satisfaction in children with autism spectrum disorder and nominated friends. J Autism Dev Disord.

[41] Weiss R (1975). Loneliness: the experience of emotional and social isolation.

[42] Dahlberg L, McKee KJ (2014). Correlates of social and emotional loneliness in older people: evidence from an English community study. Aging Ment Health.

[43] Lord C, Rutter M, DiLavore PC, Risi S, Gotham K, Bishop S (2012). Autism Diagnostic Observation Schedule.

[44] Libster N, Knox A, Engin S, Geschwind D, Parish-Morris J, Kasari C (2022). Personal victimization experiences of autistic and non-autistic children. Mol Autism.

[45] Kasari C, Dean M, Kretzmann M, Shih W, Orlich F, Whitney R, Landa R, Lord C, King B (2016). Children with autism spectrum disorder and social skills groups at school: a randomized trial comparing intervention approach and peer composition. J Child Psychol Psychiatry.

[46] Dean M, Williams J, Orlich F, Kasari C (2020). Adolescents with autism spectrum disorder and social skills groups at school: a randomized trial comparing intervention environment and peer composition. Sch Psychol Rev.

[47] Lord C, Risi S, DiLavore PS, Shulman C, Thurm A, Pickles A (2006). Autism from 2 to 9 years of age. Arch Gen Psychiatry.

[48] Parish-Morris J, Liberman MY, Cieri C, Herrington JD, Yerys BE, Bateman L, Donaher J, Ferguson E, Pandey J, Schultz RT (2017). Linguistic camouflage in girls with autism spectrum disorder. Mol Autism.

[49] Lord C, Risi S, Lambrecht L, Cook EH, Leventhal BL, DiLavore PC, Pickles A, Rutter M (2000). The autism diagnostic observation schedule—generic: a standard measure of social and communication deficits associated with the spectrum of autism. J Autism Dev Disord.

[50] Hus V, Gotham K, Lord C (2014). Standardizing ADOS domain scores: separating severity of social affect and restricted and repetitive behaviors. J Autism Dev Disord.

[51] Gotham K, Pickles A, Lord C (2009). Standardizing ADOS scores for a measure of severity in autism spectrum disorders. J Autism Dev Disord.

[52] Elo S, Kyngäs H (2008). The qualitative content analysis process. J Adv Nurs.

[53] Dedoose. (2018). Dedoose version 8.0.35, web application for managing, analyzing, and presenting qualitative and mixed method research data.

[54] Bourke B (2014). Positionality: reflecting on the research process. Qual Rep.

[55] Martin CL, Ruble DN (2010). Patterns of gender development. Annu Rev Psychol.

[56] Miller CF, Lurye LE, Zosuls KM, Ruble DN (2009). Accessibility of gender stereotype domains: developmental and gender differences in children. Sex Roles.

[57] Priess HA, Lindberg SM, Hyde JS (2009). Adolescent gender-role identity and mental health: gender intensification revisited. Child Dev.

[58] Koenig AM (2018). Comparing prescriptive and descriptive gender stereotypes about children, adults, and the elderly. Front Psychol.

[59] Hill JP, Lynch ME, Brooks-Gunn J, Petersen AC (1983). The intensification of gender-related role expectations during early adolescence. Girls at puberty.

[60] Kasari C, Rotheram-Fuller E, Locke J, Gulsrud A (2012). Making the connection: randomized controlled trial of social skills at school for children with autism spectrum disorders. J Child Psychol Psychiatry.

[61] Laugeson EA, Frankel F, Mogil C, Dillon AR (2009). Parent-assisted social skills training to improve friendships in teens with autism spectrum disorders. J Autism Dev Disord.

[62] Laugeson EA, Frankel F, Gantman A, Dillon AR, Mogil C (2012). Evidence- based social skills training for adolescents with autism spectrum disorders: the UCLA PEERS program. J Autism Dev Disord.

[63] Feldman M, Hamsho N, Blacher J, Carter AS, Eisenhower A (2022). Predicting peer acceptance and peer rejection for autistic children. Psychol Sch.

[64] Stagg SD, Thompson-Robertson L, Morgan C (2022). Primary school children rate children with autism negatively on looks, speech and speech content. Br J Dev Psychol.

[65] Fisher MH, Taylor JL (2016). Let’s talk about it: peer victimization experiences as reported by adolescents with autism spectrum disorder. Autism.

